# Reactive Arrays of Colorimetric Sensors for Metabolite and Steroid Identification

**DOI:** 10.4236/jst.2014.41001

**Published:** 2014-12-31

**Authors:** Gary Batres, Talia Jones, Hannah Johnke, Mark Wilson, Andrea E. Holmes, Sharmin Sikich

**Affiliations:** Department Chemistry, Doane College, Crete, USA

**Keywords:** Colorimetric Arrays, Sensors, Color Changes, Steroids, Metabolic Disease, Metabolites, Detection

## Abstract

The work described herein examines a rapid mix-and-measure method called DETECHIP suitable for screening of steroids and metabolites. The addition of steroids and metabolites to reactive arrays of colorimetric sensors generated characteristic color “fingerprints” that were used to identify the analyte. A color analysis tool was used to identify the analyte pool that now includes biologically relevant analytes. The mix-and-measure arrays allowed the detection of disease metabolites, orotic acid and argininosuccinic acid; and the steroids androsterone, 1,4-androstadiene, testosterone, stanozolol, and estrone. The steroid 1,4-androstadiene was also detected by this method while dissolved in synthetic urine. Some of the steroids, such as androstadiene, stanozolol, and androsterone were co-dissolved with (2-hydroxypropyl)-*β*-cyclodextrin in order to increase solubility in aqueous buffered solutions. The colorimetric arrays do not intend to eliminate ELISA or mass spectroscopy based screening, but to possibly provide an alternative analytical detection method for steroids and metabolites.

## 1. Introduction

**Figure 1:**
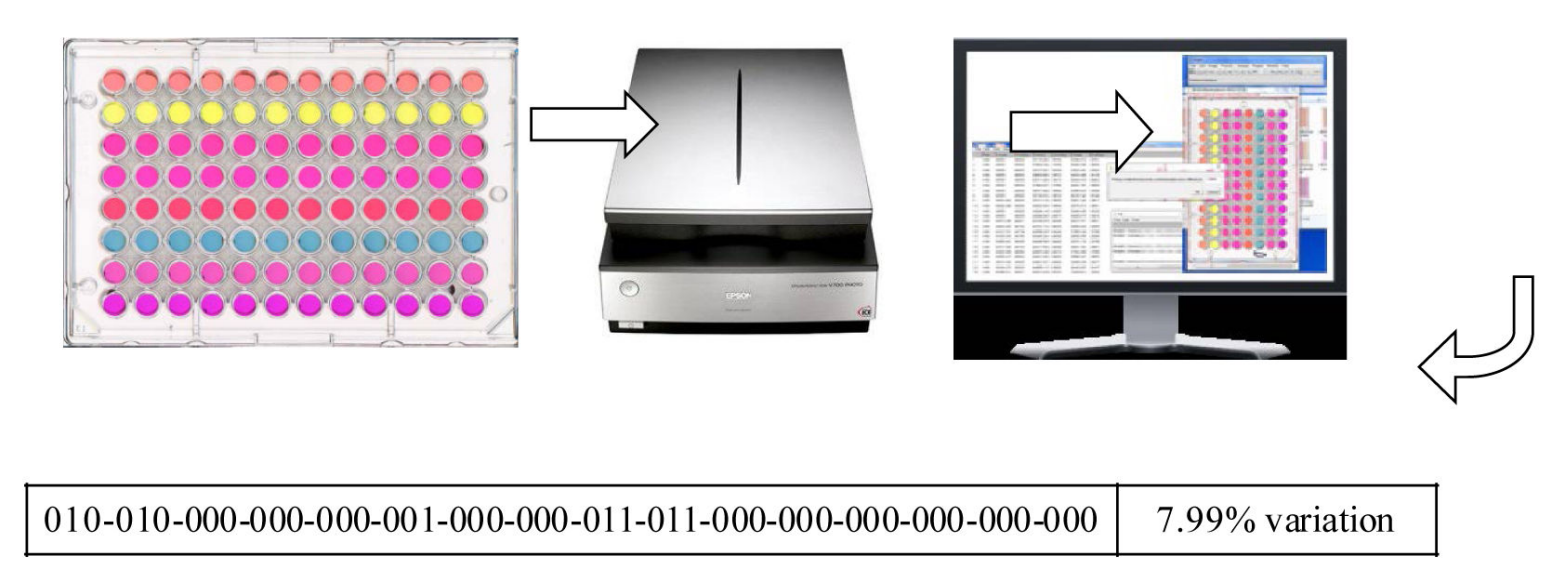
The steroid 1,4-androstadiene with (2-hydroxypropyl)-*β*-cyclodextrin was dissolved in phosphate buffer and tested in a 96-well plate DETECHIP assay. The plate is scanned by a flat bed scanner on transparency mode and then analyzed using a computer program that gives a 48-digit binary code (see bottom) for analyte identification (The % variation represents the variation from the average code).

Recent advances of reactive arrays have demonstrated convincingly that many classes of molecules can be identified in this format [1]-[6]. Our group has developed a colorimetric and fluorometric assay in a 96-well plate, DETECHIP®, which has successfully discriminated between many small molecules, with our main focus being illicit, over the counter drugs, and cutting agents. DETECHIP has color sensors DC1 - DC8 that produce a unique binary code for small molecules. The chemical structures of DC1 - DC8 are published elsewhere [3]. The binary codeis are based on color changes when the analyte-treated wells are compared to control wells [1]-[6]. The concept of DETECHIP is shown in Figure 1, where a typical assay with an analyte like 1,4-androstadiene-3,17-dione is tested. The 96-well plate is scanned by a flat-bed scanner, and the image is subjected to automated red, green, blue (RGB) analysis that renders a unique binary code for the analyte identification. A “1” in the code means that there was a color change in the individual RGB channels, where a “0” means that there was no color change in the individual RGB channels when the analyte well was compared to the control well.

We aim to expand the ability of DETECHIP detection to metabolites, both biological and synthetic, that are normally excreted in urine. Orotic acid and argininosuccinic acid are markers found in the urine of those suffering from amino acid disorders [7] [8]. Both of these are small molecules typically screened in newborns and are present in urine at low amounts when there is an excess amount of ammonia in the patient that is suffering from the amino acid disorder. Another disease commonly tested in newborns is congenital adrenal hyperplasia (CAH). CAH is a genetic steroid disorder that causes abnormal levels of steroids being produced. Androsterone and estrone levels in urine can be indicators of CAH [9] [10].

In this study, disease metabolites such as argininosuccinic acid and orotic acid, along with steroids androsterone and estrone, can be discriminated by the DETECHIP system when dissolved in water, buffer, or methanol. In addition to these metabolites, it was possible to discriminate between the anabolic steroids stanozolol, testosterone, and 1,4-androstadiene-3,17-dione. Stanozolol, for example, is an anabolic steroid also known as Winstrol. It is commonly taken by athletes to increase muscle mass. Detection and qualification of doping compounds in competitive sports are critical to ensure a fair competition. Current methods of detecting stanozolol in urine use gas chromatography-mass spectrometry (GCMS) or liquid chromatography-mass spectrometry (LCMS) [11] [12].

Despite advances in detection systems, there is still a lack of actual applications of simple colorimetric arrays (that simply change color when exposed to analyte without the use of antibodies) used to detect biological samples dissolved in urine samples [13]-[19]. The main challenge of colorimetric arrays is the fact that the analyte that is most abundant is usually detected, and analytes that are less abundant and that may be the disease marker are not detected and overshadowed by the component that has a higher concentration in the mixture. In this study, we also show that DETECHIP will produce a unique detection signature for the anabolic steroid 1,4-androstadiene-3,17-dione when it is dissolved in synthetic urine.

## 2. DETECHIP®

### 2.1. Methods

DETECHIP 96-well plate assays were prepared as previously published with eight sensors DC1 - DC8, in order to test the efficacy of DETECHIP for distinguishing steroids and metabolites over a range of analyte concentrations [1] [5]. All reagents (except for the synthetic urine) were purchased from Sigma Aldrich. The synthetic urine was purchased from Ricca Chemical Company (Cat. No. 8361-1).

### 2.2. Preparation of Metabolites and Steroid

#### Preparation of 1,4-Androstadiene

A solution of 12 mM 1,4-androstadiene was dissolved in synthetic urine with (2-hydroxypropyl)-*β*-cyclodextrinat a mass:mass ratio of 1(1,4-androstadiene):9 (b-cyclodextrin). The *β*-cyclodextrin was added first to the synthetic urine and stirred until it was completely dissolved. The 1,4-androstadiene was added slowly to the *β*-cyclodextrin and urine solution. The solution was stirred overnight until it was completely dissolved. A control solution without 1,4-androstadiene was made for the 96-well plate by dissolving the same amount of (2-hydroxypropyl)-*β*-cyclodextrin in urine. A 96-well plate was prepared as previously published using the *β*-cyclodextrin as the control and 12 mM 1,4-androstadiene with *β*-cyclodextrin in urine as the analyte [2].

#### Preparation of Stanzolol Solutions

A solution of 12 mM of stanozolol was dissolved along with (2-hydroxypropyl)-*β*-cyclodextrin in a 1:9 mass ratio (steroid: cyclodextrin) in a 400 mM phosphate buffer at pH 7. The (2-hydroxypropyl)-*β*-cyclodextrin was added first to the buffer. The solution was stirred until dissolved. The stanozolol was added slowly and the solution was stirred and heated to about 65°C - 70°C without boiling until the entire steroid dissolved. A control solution was made by dissolving *β*-cyclodextrinalone at the same concentration in 400 mM phosphate buffer at pH 7. The 96-well plate was made by pipetting 150 μL of 400 mM Tris buffer at (pH 7) into columns 1, 2, 5, 6, 9, and 10. 150 μL of 400 mM phosphate buffer at (pH 7) was pipetted into columns 3, 4, 7, 8, 11, and 12. Then, 30 μL of DC2 was pipetted into rows 1 and 4. This was repeated for DC4, but using rows 2 and 5, and DC8 was pipetted into rows 3 and 6. Next, 120 μL of the control solution containing the *β*-cyclodextrin and phosphate buffer was added to columns 1, 3, 5, 7, 9, and 11. Finally, 120 μL of the 12 mM stanozolol solution with *β*-cyclodextrin was pipetted into columns 2, 4, 6, 8, 10, and 12. This makes a total of six replications on one plate.

#### Preparation of Argininosuccinic Acid, Orotic Acid, Androsterone and Estrone

Argininosuccinic acid was prepared at 9.25 mM in water while orotic acid was prepared at the lower concentration of 6 mM due to its low solubility in water. A solution of 6 mM androsterone was dissolved in water using a 1:12 mass ratio with (2-hydroxypropyl)-*α*-cyclodextrin. The same procedure for making the assays was followed for androsterone except *α*-cyclodextrin was used in this case to help solubilize the steroid and the control solutions containing the same concentration of *α*-cyclodextrin in water as there was in the analyte solution. Androsterone and estrone were also dissolved in methanol because of their low solubility in water. For the methanol-dissolved androsterone and estrone only assays, DC1-DC8 were also prepared using methanol (750 μM).

### 2.3. Analyzing 96-Well Plates Using Image to Obtain a Binary Code

**Figure 2:**
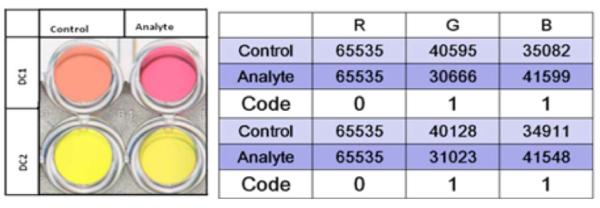
Left—This image shows a visible color change in DC1 but not in DC2. Right—This table shows the resulting code for the given image after RGB analysis. The RGB values in the table represent the total red, green, or blue value for all the pixels in a set area of each well in the image. For DC2, the image analysis detects color change (as indicated by differences in the total color value) in the green and blue channels that the human eye cannot see.

The 96-well plate was scanned using an Epson Perfection V700 photo flatbed scanner. The settings for the scanner were Film (with Film Area Guide) document type, Positive Film type, 48-bit Color, 400 dpi resolution, 8.00 × 10.00 inches document size, and Unsharp Mask on. An RGB analysis was then performed using an in-house designed macro and Image J (http://rsb.info.nih.gov/ij/) software. The change in red, green, and blue values was examined above a given threshold of 1000. A “1” was given for each change in total value above the given threshold. A “0” was given for no change in total value below the threshold (see Figure 2). The percent variation is determined by analyzing the frequency of the average code. The most frequent code is determined for an individual color change, either with a “0” or “1”. Then, the amount of the lesser frequent code is determined. The amount of the lesser frequent code is subtracted from the amount of the more frequent code. This difference is divided by the number of trials and converted to percentage.

## 3. Discussion of Results

RGB analysis of DETECHIP has been very successful in determining the identity of analytes. As seen in Figure 2, an excerpt of a 96-well DETECHIP plate demonstrates a vivid visible color change in DC1 when the control well and the analyte well are compared. The RGB table shows three RGB numbers for the control wells and the RGB values for the analyte well. The red channel of the control is identical to the analyte where the green and blue channels show significant changes between the control and the analyte. Therefore, the red channel renders a code of “0” where as the blue and green channels each render a code of “1”. Although DC2 does not show a visible color change, RGB analysis demonstrates similar color changes in the green and blue channel giving the same code as in DC1. As human color perception varies, the RGB analysis of scanned images via computer is more objective and less susceptible to human error, and detects changes in individual color that the human eye may not be able to detect.

Table 1 shows the different binary codes obtained using RGB analysis for the analytes tested for this study. All the codes show a unique combination of “0” and “1”, which means that DETECHIP can be used to discriminate between these analytes. The percent variation shows the variation in the code between the trials and the lower the variation, the more reliable is the reproducibility of the code. The threshold represents the predefined difference between the control and analyte total color value that has to be overcome in order for a color change to be registered as a “1”. The threshold of 1000 was optimal for these experiments as it rendered enough sensitivity for each analyte. A lower threshold of 500 resulted in too many “1”s and a higher threshold of 2000 rendered too many “0”s.

Some of the analytes were not soluble in aqueous buffered solution. Therefore, androsterone was dissolved into solution using (2-hydroxypropyl)-*α*-cyclodextrin whereas 1,4-androstadiene was dissolved in urine using (2-hydroxypropyl)-*β*-cyclodextrin. The androstadiene was not soluble enough in *α*-cyclodextrin; but due to structural differences between the two cyclodextrins, the *β*-cyclodextrin worked better to dissolve the steroid. Other solvents like methanol were used to dissolve androsterone and estrone (last two entries in Table 1). When analytes were dissolved in methanol, DC1-DC8 were also dissolved in methanol; consequently reducing the binary code in half, because of the elimination of the two-buffer system.

As seen in Table 1, the lowest percent variation of 7.99% was found for 1,4-androstadiene in urine. The highest percent variation of 19.81% was for argininosuccinic acid in water. Eliminating dyes that show a lot of variation in the code can reduce the high error. After indentifying and eliminating these dyes, a test was created that gives clear signature color changes for stanozolol. The code for stanzolol in DC2, DC4, and DC8 is shown in Table 2. This result shows that certain sensors can be isolated from the DETECHIP platform and used for specific detection of certain analytes. This could open more practical aspects of DETECHIP because if assays are developed using only two or three sensors versus eight or more, miniaturization and code analysis may be less complex.

It is noted that current concentrations do not reflect the actual concentrations that occur in biological samples. However, the technology will be improved by miniaturization of DETECHIP to convert the 96-well plate assay into a microarray that is smaller than a fingernail. This miniaturization will allow for testing of analytes at much lower concentrations because smaller amounts of the sensors will require smaller amount of analyte.

In conclusion, we have shown that DETECHIP technology can be used to detect more diverse molecules besides the original target analytes of drugs and cutting agents. The expansion of analytes to metabolites, disease markers, and steroids widens the scope of this method and could potentially be relevant in biomedical applications.

Future studies will include the analysis of more amino acids and other steroids in urine, including further testing of stanozolol and androsterone that we presented in this paper.

## Figures and Tables

**Table 1:** Binary codes for metabolites and steroids tested in this study using the DETECHIP system.

Analyte Information	RGB Code	Percent Variation
Orotic Acid 6 mM in water 1000 threshold N = 9	000-010-001-110-000-001-001-001-000-000-110-111-000-101-000-101	15.1%
Argininosuccinic acid 9.25 mM in water 1000 threshold N = 9	001-000-001-001-000-000-000-000-000-001-111-111-000-000-000-101	19.81%
Argininosuccinic acid 6 mM in water 1000 threshold N = 3	000-000-000-000-000-000-000-000-000-000-100-111-000-000-000-000	4.16%
1,4-Androstadiene 12 mM (2-hydroxypropyl)-*β*- cyclodextrin 1:9 mass ratio in urine 1000 threshold N = 6	010-010-000-000-000-001-000-000-011-011-000-000-000-000-000-000	7.99%
Androsterone 6 mM (2-hydroxypropyl)-*α*-cyclodextrin 1:12 mass ratio in water 1000 threshold N = 3	011-011-001-001-011-011-001-001-011-011-111-111-001-101-001-101	15.04%
Androsterone 12 mM in methanol 1000 threshold N = 6	000-000-001-000-011-111-011-001	17.82%
Estrone 12 mM in methanol 1000 threshold N = 6	000-000-000-000-011-100-011-000	12.96%
Stanozolol 12 mM in phosphate buffer and cyclodextrin N = 9	011-011-110-110-011-001-001-001-011-011-011-011-101-101-101-101	10.97%
Testosterone 12 mM in phosphate buffer and cyclodextrin N = 9	011-011-001-000-001-001-001-001-010-010-101-000-000-000-000-000	15.85%

**Table 2:** A specific DETECHIP assay has been developed for Stanzololusing DC2 in Buffer A, and DC4 and DC8 in buffers A and B. There is a visual color change in DC2, DC4, and DC8 that is characteristic for stanozolol and very different from testosterone in the same assay.

Steroid, concentration, solvent, threshold, N = number of trials	Binary code	Percent Variation
Stanozolol 12 mM in water 1000 threshold N = 6	111-001-001-101-101	5.56%
Stanozolol 12 mM in water 2000 threshold N = 6	110-001-001-101-101	1.11%
Testosterone 12 mM in phosphate buffer N = 9,1000 threshold	001-001-001-000-000	2.59%
Testosterone 12 mM in phosphate buffer N = 9,2000 threshold	000-000-000-000-000	0%
